# matRadiomics: A Novel and Complete Radiomics Framework, from Image Visualization to Predictive Model

**DOI:** 10.3390/jimaging8080221

**Published:** 2022-08-18

**Authors:** Giovanni Pasini, Fabiano Bini, Giorgio Russo, Albert Comelli, Franco Marinozzi, Alessandro Stefano

**Affiliations:** 1 Institute of Molecular Bioimaging and Physiology, National Research Council (IBFM-CNR), Contrada Pietrapollastra-Pisciotto, 90015 Cefalù, Italy; 2 Department of Mechanical and Aerospace Engineering, Sapienza University of Rome, Eudossiana 18, 00184 Rome, Italy; 3 Ri.MED Foundation, Via Bandiera 11, 90133 Palermo, Italy

**Keywords:** radiomics, software package, machine learning, image analysis, PET, MRI, CT

## Abstract

Radiomics aims to support clinical decisions through its workflow, which is divided into: (i) target identification and segmentation, (ii) feature extraction, (iii) feature selection, and (iv) model fitting. Many radiomics tools were developed to fulfill the steps mentioned above. However, to date, users must switch different software to complete the radiomics workflow. To address this issue, we developed a new free and user-friendly radiomics framework, namely matRadiomics, which allows the user: (i) to import and inspect biomedical images, (ii) to identify and segment the target, (iii) to extract the features, (iv) to reduce and select them, and (v) to build a predictive model using machine learning algorithms. As a result, biomedical images can be visualized and segmented and, through the integration of Pyradiomics into matRadiomics, radiomic features can be extracted. These features can be selected using a hybrid descriptive–inferential method, and, consequently, used to train three different classifiers: linear discriminant analysis, k-nearest neighbors, and support vector machines. Model validation is performed using k-fold cross-Validation and k-fold stratified cross-validation. Finally, the performance metrics of each model are shown in the graphical interface of matRadiomics. In this study, we discuss the workflow, architecture, application, future development of matRadiomics, and demonstrate its working principles in a real case study with the aim of establishing a reference standard for the whole radiomics analysis, starting from the image visualization up to the predictive model implementation.

## 1. Introduction

The development of several image segmentation algorithms, and the use of artificial intelligence (AI) in the form of deep learning or machine learning provided clinicians with a new set of tools for performing medical images analysis [1]. Typically, a single portion of the medical image is identified as a target from which to extract quantitative metrics. Such data extracted from a patient dataset can be used to build predictive models.

In such a way, a rapidly evolving new research field called radiomics [2] wraps the different computational techniques into a workflow consisting of several crucial steps: (i) the target is identified through manual image inspection or automatic identification; (ii) the target is segmented using manual, semi-automatic or automatic algorithms; (iii) the radiomic features are extracted from the target; (iv) the features are reduced and selected; and (v) the selected features are used to build predictive models [3,4].

A major advantage of radiomics data is that they are mineable, so they can be used to identify personalized predictive and/or prognostic models to support the medical decision process. Radiomic features can be used to verify the characteristics of a lesion and its evolution over time, potentially capturing the evolution of the disease and improving the prediction of overall survival and/or patient outcome [5,6]. Radiomic features are commonly grouped into three large classes: shape features, or morphological features; first order statistical features and texture features. The goal of shape features is to describe geometric characteristics of the volume of interest (VOI), such as the mesh total volume and total surface, while first order statistical features are used to describe the grey level distribution within the VOI, such as kurtosis and skewness. The texture features, which are the most complex, are instead used to obtain information about the grey level patterns within the VOI.

To perform the feature extraction task, many software and radiomics computational frameworks were developed, with LIFEx [7] and Pyradiomics [8] being two of the most popular. LIFEx is an image biomarker standardization initiative (IBSI) [9] and compliant freeware that allows the user to complete the first three steps of the workflow, from target identification to feature extraction. IBSI addresses the main issue in the feature extraction process: the reproducibility of the extracted features. For this reason, the IBSI was introduced for the standardization of radiomic features. LIFEx can be used to interact with its user interface (UI). On the other hand, Pyradiomics is an open-source IBSI -compliant Python package that can only be used to perform feature extraction and does not have its own UI. Its advantage is that it can be integrated into other software solutions, such as 3D Slicer [10], which works like Pyradiomics UI.

However, to date, most radiomics software available online stops at the feature extraction step, not allowing the user to complete the radiomics workflow. Often, the clinician and/or the researcher must switch to external software to perform feature selection and machine learning. For example, Pyradiomics can only be used for the feature extraction process, while LIFEx integrates segmentation algorithms and the feature extraction process. Among the other software available, MAZDA is a framework based on C++/Delphi [11] that can perform all radiomics tasks, but it is not IBSI compliant, nor macOS or open source compatible. Meanwhile, FeAture Explorer (FAE) [12] does not include segmentation algorithms. Furthermore, all the mentioned radiomics frameworks do not integrate a data harmonization method necessary in multicenter studies. As is well known, radiomic features can show batch effects when several image scanners are involved [13,14,15].

To address these issues, we developed a new freeware and IBSI compliant computational radiomics frameworks with graphical user interface (GUI), namely matRadiomics, which allows users to complete the whole radiomics workflow within the same software, simplifying the radiomics process and focusing on result reproducibility thanks to metadata that tracks the matRadiomics configuration options set by the user. matRadiomics is based on MATLAB [16] and Python [17], supports both macOS and Windows operating systems, includes an innovative algorithm for feature selection, has a user-friendly interface and integrates Pyradiomics. Furthermore, matRadiomics can be distributed both in the compiled version (Standalone MATLAB Application) and in the non-compiled one. The advantage of the compiled version is that it does not need MATLAB to work, but MATLAB Runtime, which does not require a license to be used, thus making matRadiomics suitable for use in clinical practice.

Furthermore, matRadiomics integrates ComBat, one of the best known methods for feature harmonization [13,14,15].

In this study, we discuss the workflow, architecture, application, and future developments of matRadiomics, and demonstrate its working principles in a case study derived from the Lung Image Database Consortium (LIDC-IDRI) [18], with the aim to discriminate between benign and malignant lung nodules. The source code, documentation, and examples are available upon request to the authors. After the publication of the article, the software will be available on the authors’ institutional websites with the aim of establishing a reference standard for radiomics analyses though a freeware tool for the scientific community.

## 2. Platform

**Figure 1:**
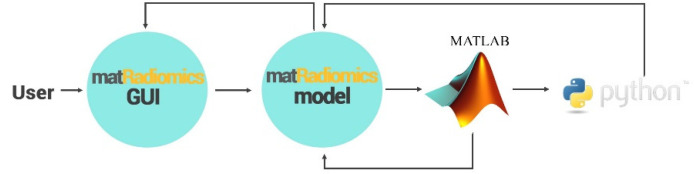
matRadiomics Architecture.

matRadiomics is a computational radiomics framework that allows the user to complete the whole radiomics workflow. Its architecture is shown in Figure 1. The main operations that can be performed are: (i) create or import a radiomics study; (ii) the import and visualization of DICOM (Digital Imaging and Communication in Medicine) images and metadata; (iii) segmentation of the target, (iv) importing of external segmentations (e.g., DICOM in Medicine-Radiation Therapy, namely DICOM-RT); (v) feature extraction using Pyradiomics; (vi) feature selection using a new hybrid-inferential descriptive algorithm [19]; (vii) machine learning algorithms.

### 2.1. Architecture

The matRadiomics platform can perform radiomics studies based on medical image datasets, such as computed tomography (CT),positron emission tomography (PET), and magnetic resonance imaging (MRI), using a user-friendly GUI. The main code is written in MATLAB while some ad-hoc features are implemented using Python. Ad-hoc functionalities are called main code by MATLAB to complete specific tasks, such as importing DICOM images, feature extraction, and machine learning. The output of the matRadiomics model is returned to the matRadiomics GUI. Three MATLAB toolboxes are used: the image processing toolbox, as well as the statistics and machine learning toolbox.

Ad-hoc functionalities are implemented within three modules: (i) the dicomModule; (ii) the pyradiomicsModule; and (iii) the classificationModule.

The dicomModule consists of two functions: the first one is used to parse and store all the DICOM attribute names, tags, and VR (value representations) types in lists; the second one is used to obtain some DICOM attribute values (ex. the slice location, rescale intercept and rescale slope) needed for further operations.

The pyradiomicsModule consists of a single function, the purpose of which is to configure the pyradiomics extractor with the settings chosen by the user.

The classificationModule consists of as many functions as the number of implemented classifiers in matRadiomics. It is used to perform model training, cross validation, and to obtain the model performance metrics.

**Figure 2:**
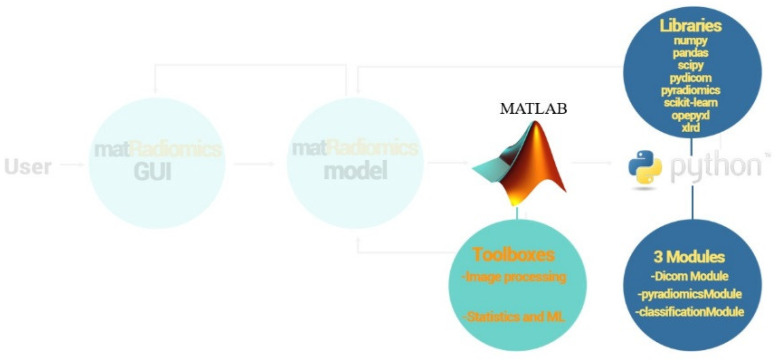
MATLAB toolboxes, python modules, and libraries.

Figure 2 shows the MATLAB toolboxes, the Python Modules, and libraries used in matRadiomics.

### 2.2. Image Visualizazion

Each matRadiomics session begins with the creation of a new radiomics study. This procedure sets the root folder where the results of the extraction, selection, and machine learning process are saved. Single frame DICOM images are imported thanks to the dicomModule based on the Pydicom library. During the import procedure, some operations are performed, such as grey levels rescaling from discrete values to common units (e.g., Hounsfield unit (HU) for CT images), storing DICOM metadata, sorting images, and the extraction of the scan main orientation and anatomical directions. The images shown are interpolated using a bilinear algorithm (property of the MATLAB Image Object). Note that the interpolation is performed only for visualization.

### 2.3. Segmentation of the Target

The target can be segmented using manual and semi-automatic segmentation algorithms. In the first case, the target contours can be manually drawn slice by slice to delimitate the volume of interest (VOI). A mask automatically fills the delimitated area. The mask can be corrected manually using an erase tool. The semi-automatic segmentation that can be used consists of a thresholding method that uses a percentage of the maximum level of grey in the VOI as a threshold. The percentage can be set manually by the user. Segmentations consisting of a single frame or a multi frame file can also be imported.

### 2.4. Radiomics Feature Extraction, Hamonization and Selection

matRadiomics integrates the Pyradiomics extractor whose options can be fully customized thanks to its user-friendly GUI. The user can select which features to extract. The features extracted for each patient are automatically saved in the study folder.

In the case of multi-center studies, matRadiomics integrates the MATLAB ComBat package to perform feature harmonization [13,14,15].

**Figure 3:**
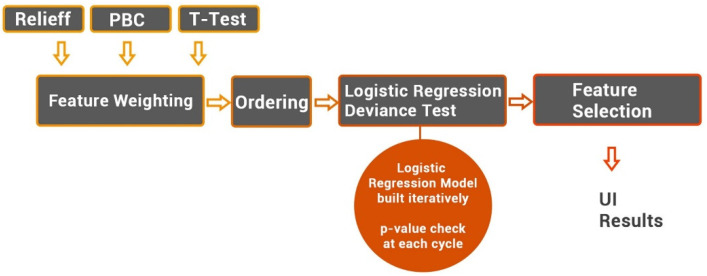
Feature selection workflow.

After the feature extraction process, and the harmonization, if used, the reduction and selection process can be started. Specifically, two feature selection approaches can be chosen. The first one consists of a hybrid-inferential descriptive algorithm that uses a Point Biserial Correlation (PBC) to assign scores to features, order them by score, and then iteratively build a logistic regression model, as extensively reported in [19]. Briefly, at each cycle, the *p*-value of the model is compared with the *p*-value of the previous cycle. If the *p*-value does not decrease at the current cycle, the procedure stops, and the logistic regression model is obtained. Furthermore, two other algorithms were implemented to assign scores to features instead of PBC. These are the *t*-test [20], and the Relieff algorithms [21] (see Figure 3).

The second one is LASSO (least absolute shrinkage and selection operator), implemented through the MATLAB lassoglm function. This method is suitable for high-dimensional data [22]. The LASSO regularization is cross validated, and the minimum deviance criterion is used to select the lambda regularization parameter. The number of folds used in cross validation is selected by the user.

**Figure 4:**
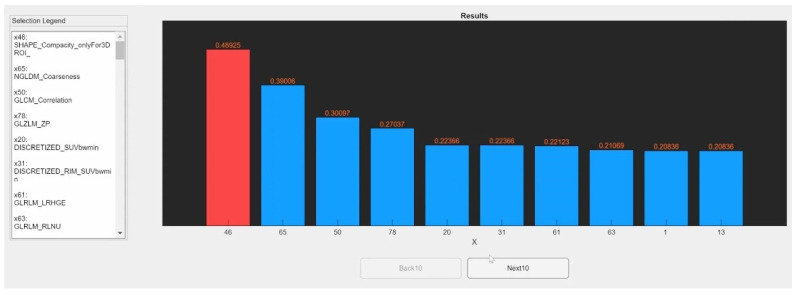
Feature selection result example. Bar plot with scores assigned to features. On the left the legend. Features selected are shown in red.

Once the selection process is complete, the results are displayed in the matRadiomics GUI. For the first approach, the features are sorted by score, and the selected features are shown in red. A legend linking the number of the feature to its name is shown on the left of the matRadiomics GUI. An example is provided in Figure 4.

For both approaches, the radiomics signature is showed as the sum of the product of the selected features and their corresponding coefficient, obtaining the so-called radiomics score (radscore).

### 2.5. Machine Learning

The classification module is used to build the predictive model. The Python scikit-learn library was used to implement functions that allow model validation, model training, and model performance evaluation. When the classification module is called, (i) the dataset is divided into training and test sets (the user chooses the test set by interacting with the matRadiomics GUI), (ii) the model is cross validated, and (iii) the model is tested using the test set. While K-fold cross validation and K-fold stratified cross validation are used to perform model validation, linear discriminant analysis (LDA) [23], K-nearest neighbors (KNN) [24], and support vector machines (SVM) [25] are used to build the radiomics model. The aim of the classification module is to obtain performance metrics, such as the accuracy, true positive rate, and false positive rate for each model produced by the K-fold cross-validation. Then, these metrics are sent to the matRadiomics model to build receiver operating characteristic (ROC) curves and display the results.

**Figure 5:**
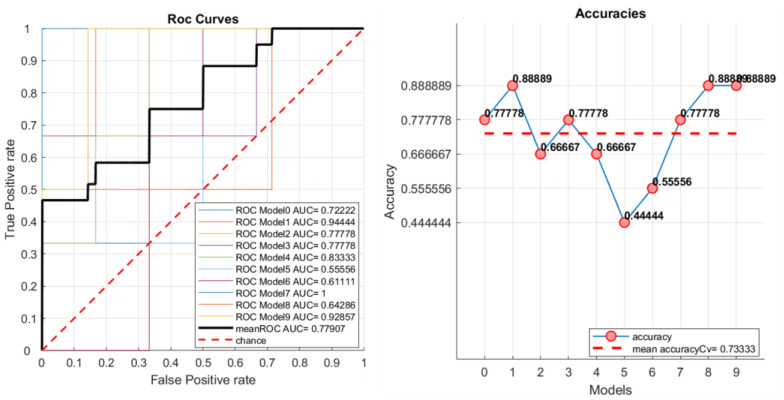
Example of ROC curves, AUCs, and accuracies.

**Figure 6:**
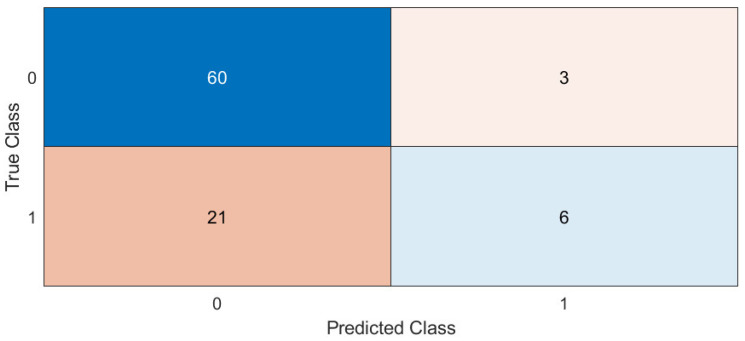
Example of the total confusion matrix.

The average ROC curve is computed as the global performance metric. It is calculated as an average of the ROC curves generated for each k group produced by the cross validation. Moreover, area under curve (AUC) values are computed for each ROC and for the average ROC. The accuracy, mean accuracy, and total confusion matrix are displayed in the matRadiomics GUI. Examples are shown in Figure 5 and Figure 6. Finally, to assess the model robustness, the model performance metrics can be calculated on the test set (if this is not empty), and the resulting metrics are shown in the matRadiomics GUI, together with the validation metrics.

### 2.6. Import an External Radiomics Study

To enable active collaboration between researchers, matRadiomics allows the user to import external studies to increase the number of patients in the original dataset, or simply, to divide the work among multiple users. In this way, features extracted from other matRadiomics users can be imported and new patients can be automatically elaborated using the same setting. Therefore, a study can be shared and matRadiomics automatically configures itself using the metadata saved in the study folder. The metadata consist of a json file that contains several attributes that track each option set by the user during each step of the radiomics workflow. For example, they track which type of images were used (ex. MRI, PET, CT), which segmentation algorithm was used, and the options used to configure the Pyradiomics extractor.

## 3. Case Study

matRadiomics was used to perform all steps of the radiomics workflow in a case study. The aim was to implement a predictive model capable of discriminating between benign and malignant lung nodules. Moreover, we evaluated the impact of the feature harmonization on the results obtained using Combat, integrated in matRadiomics, as reported in Section 2.4.

### 3.1. The Dataset

The dataset was derived from the LIDC-IDRI [18], which consists of lung cancer screening and diagnostic CT scans. From the LIDC-IDRI dataset, 93 cases (29 benign and 64 malignant) were included in our study because the diagnosis was known. Due to the limited number of patients and the lack of external validation, the test set was not created and only k-fold cross validation was performed: the results obtained by averaging the results of all k-folds are more robust than those obtained on a very small test set [26].

The DICOM images and the segmentations were imported into matRadiomics and the correct overlapping of the segmentations on the images was checked. For one patient, a segmentation did not overlap the lung nodule, so the patient was excluded from the dataset. We concluded with a total number of patients equal to 92 (28 benign, 64 malignant).

As the LIDCI-IDRI dataset collects scans that come from multicenter studies, differences in scanner models, pixel spacing, and slice thickness were found. We investigated their values as reported in Table 1, Table 2 and Table 3. The matrix dimensions [rows, col] were [512, 512] for all studies.

### 3.2. Feature Extraction

The lack of a practical guidelines suggesting how to set extraction options is currently a major issue in radiomics [27]. Therefore, we referred to a scientific paper that reported a study carried out on the same dataset (LIDC-IDRI) to set the value of the bin size [28]. As a result, we set it equal to 64, while we left the Pyradiomics default settings for all other extraction options. Finally, we obtained 107 features. Then, using the matRadiomics user interface, we labelled each patient as a benign or malignant nodule. Patients diagnosed with benign lesion were labelled 1, while those diagnosed with malignant lesion were labelled 0.

### 3.3. Feature Harmonization

The aim of this step is to verify if difference in scanner models influences the extracted features and, consequently, the selection and machine learning results.

As shown in Table 1, only one case belonged to batch six. Therefore, we excluded it from the derived dataset. We obtained a dataset of 91 cases (28 benign, 63 malignant).

**Figure 7:**
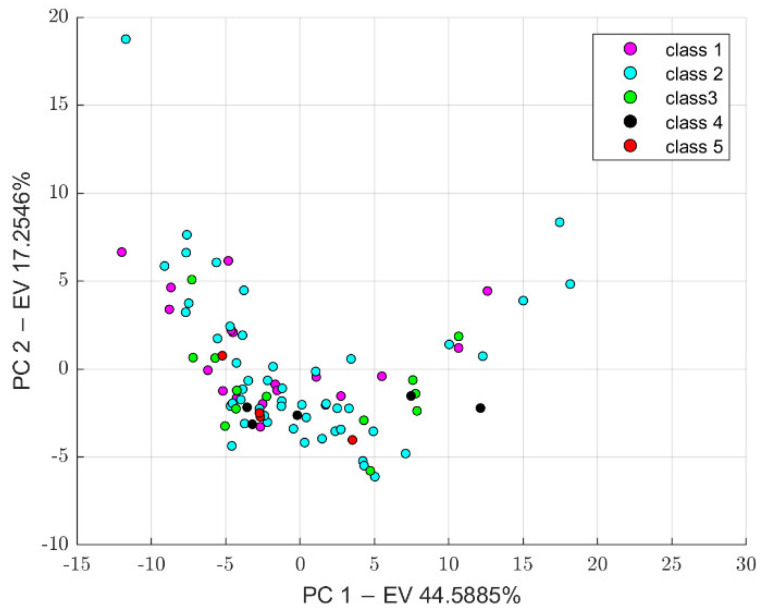
PCA results, PC 1: First Principal Component, PC 2: Second Principal Component, EV: Explained Variance.

**Figure 8:**
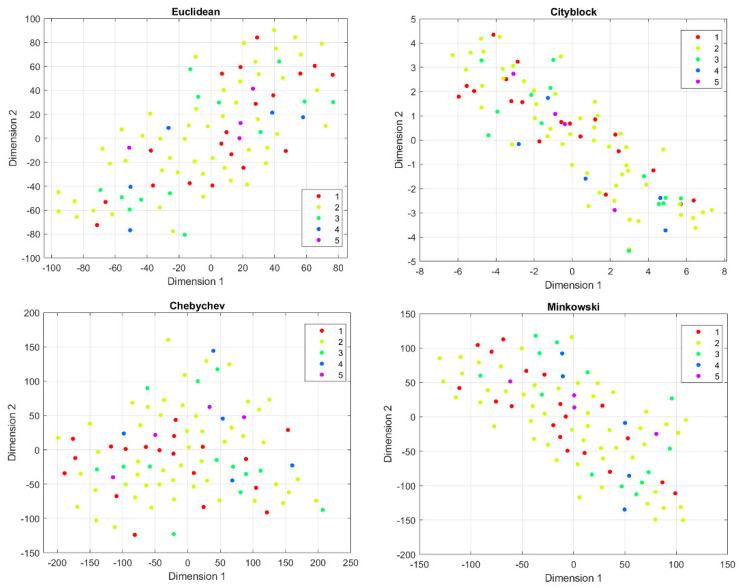
t-SNE results for different distance measurements. (**top left**): Euclidean distance, (**top right**): cityblock distance, (**bottom left**): chebychev distance, (**bottom right**): minkowsi distance.

**Figure 9:**
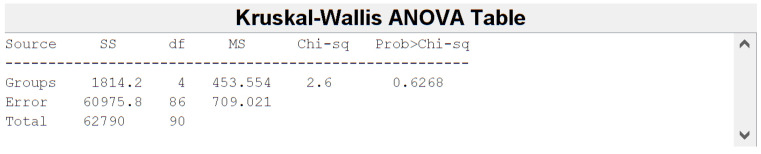
Kruskal-Wallis Table for the PC 1 scores. *p*-value = 0.6268 > 0.05.

**Figure 10:**
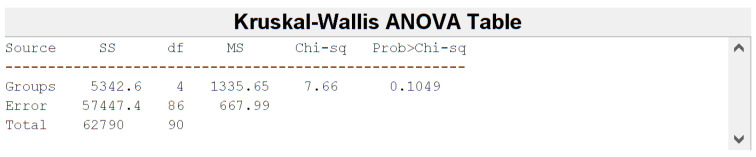
Kruskal-Wallis Table for the PC 2 scores. *p*-value = 0.1049 > 0.05.

**Figure 11:**
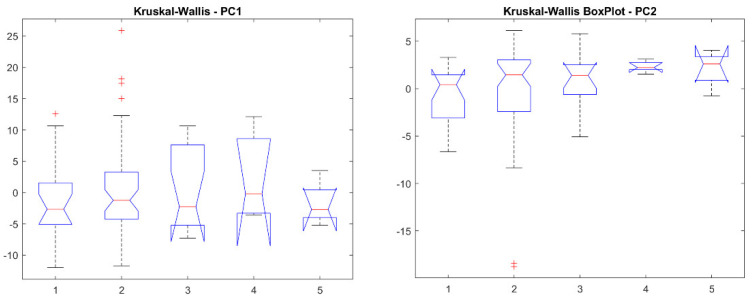
Box plots for the PC1 and PC2 scores.

Before feature harmonization, we verified if the extracted features were affected by a batch effect. Thus, principal component analysis (PCA) was performed on the extracted features to plot data in a space of reduced dimensions. Visual inspection of Figure 7 suggests the absence of batch effects. Furthermore, we used t-distributed stochastic neighbor embedding (tSNE) to check for batch effects using four different distance methods: Euclidean, cityblock, minkowski, and chebychev. Again, the results shown in Figure 8 suggest the absence of clusters. Finally, the Kruskal–Wallis test, carried out on both the first and second main component scores, also confirmed the absence of clusters (*p*-value threshold = 0.05, null hypothesis: data in each group comes from the same distribution). The results of the statistical analysis are shown from Figure 9, Figure 10 and Figure 11.

Although the analysis confirmed the absence of batch effects, we decided to harmonize the features using the ComBat package integrated in matRadiomics to evaluate its correct functioning. Therefore, two datasets, the non-harmonized and the harmonized one, were considered for all further analyses (feature selection, machine learning).

### 3.4. Feature Selection

The feature selection was performed to reduce and select the extracted features as reported in Section 2.5. The procedure was repeated for the non-harmonized features and for the harmonized features. In both cases, point biserial correlation was chosen as the method for assigning scores to features. As a result, the selection process identified the same feature for both groups, as shown in Table 4.

The matRadiomics results confirm that the harmonization does not influence the selected feature and corroborate the hypothesis of the absence of batch effects due to different scanner models.

Moreover, the feature selection process was repeated using the LASSO algorithm for the non-harmonized group, as shown in Table 5.

### 3.5. Machine Learning

As per the first analysis, the feature selected using the hybrid-descriptive inferential method was used to train the LDA classifier. K-fold cross validation (k-fold = 10) was used to validate the model [17]. To overcome the dataset imbalance, the training was repeated using a k-fold stratified cross validation (k-fold = 10). Before the training process started, the dataset was automatically shuffled. Therefore, we repeated the training 10 times for both validation methods. We obtained mean AUC, mean accuracy, mean sensitivity, and mean specificity. All results are reported by expressing the mean and the 95% confidence interval. The results for the k-fold cross validation are shown in Table 6, while the results for the k-fold stratified cross validation are shown in Table 7.

**Figure 12:**
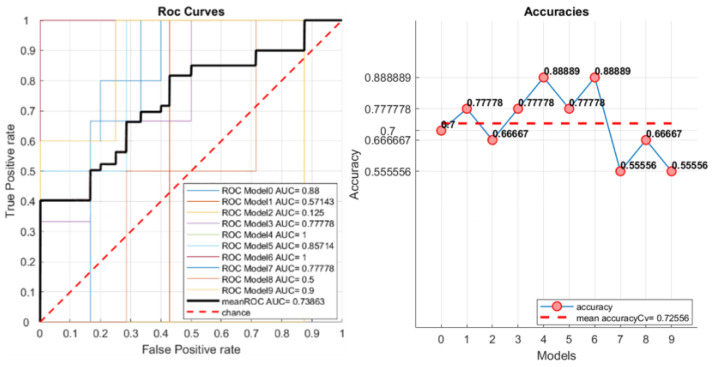
ROC curves and accuracies examples for the non-harmonized group.

Figure 12 shows an example of the ROC curves and accuracies obtained for the non-harmonized group.

Secondly, the LDA classifier was trained using the features selected by the LASSO algorithm. In this case, only a k-fold stratified coss validation (k = 10) was used to validate the model. The results are shown in Table 8.

## 4. Discussion and Conclusions

The focus of this study was to show the potential of matRadiomics in carrying out all the steps of a radiomics workflow. matRadiomics is a complete radiomics framework with a user-friendly GUI, allowing the import of DICOM images, to visualize their metadata, to segment the target, to perform feature extraction, feature harmonization and selection, and machine learning within the same software. It is mainly based on MATLAB [16] because some MATLAB-based segmentation algorithms were implemented by our IT research group [24,29,30,31,32,33,34,35] will be integrated in the next versions. Furthermore, it integrates the IBSI compliant Pyradiomics framework to fulfill the feature extraction step, Combat to perform the feature harmonization process, and an innovative hybrid method [19] for feature selection. matRadiomics is meant to be used intensively to produce a large amount of data with the aim of supporting clinicians in medical diagnosis. Moreover, it uses metadata to track all settings to improve reproducibility when data are shared.

One of the main limitations of the software, which will have to be overcome in future releases through the integration of GridSearch and Randomized Search [36], is the lack of automated hyperparameter optimization methods. Currently, all hyperparameters, except the number of k neighbors used by the KNN classifier, are set as default to avoid an overly complicated matRadiomics GUI for the end-user.

Another limitation is the lack of a fixed and automatic radiomics pipeline that allows the user to import images and directly obtain the final model. In our case, the user must supervise every single radiomics step to avoid any error, both during the target segmentation phase and in the setting of the parameters of the feature extraction process. To date, this is the major issue in all radiomics studies involving machine learning algorithms due to the lack of standardization in both segmentation and the feature extraction processes. To overcome these issues, deep learning based radiomics models were proposed. Therefore, the tumor delineation process is automatic and there is no need to set parameters for feature extraction. Using deep learning algorithms, the process can be reduced to a single automated process that only needs images as input. However, these models require a large amount of data to be properly trained and avoid overfitting, a characteristic difficult to achieve in biomedical imaging studies where datasets are usually very small [37].

Finally, as a further development, matRadiomics will be improved by adding new automatic and semi-automatic segmentation algorithms, as already reported above [24,29,30,31,32,33,34,35], support for more file formats (e.g., NIfTi) [38], more advanced harmonization methods [39], and co-registration of multi-modal images [40].

In conclusion, by providing this innovative radiomics platform, we aim to establish a reference standard for whole radiomics analyses starting from image visualization to predictive model implementation.

## Figures and Tables

**Table 1:** Different Scanner Models.

Scanner Model	Total	#Benign	#Malignant	Batch ID
LightSpeed VCT (GE)	19	5	14	1
LightSpeed16 (GE)	50	19	31	2
LightSpeed Plus (GE)	13	4	9	3
LightSpeed Pro 16 (GE)	5	0	5	4
LightSpeed Ultra (GE)	4	0	4	5
LightSpeed Power (GE)	1	0	1	6

**Table 2:** Pixel Spacing.

Pixel Spacing [x, y] mm	Total	#Benign	#Malignant
[0.585938, 0.585938]	2	0	2
[0.605469, 0.605469]	1	0	1
[0.625000, 0.625000]	4	1	3
[0.644531, 0.644531]	3	0	3
[0.664062, 0.664062]	5	2	3
[0.683594, 0.683594]	1	0	1
[0.703125, 0.703125]	27	7	20
[0.722656, 0.722656]	3	2	1
[0.732422, 0.732422]	1	1	0
[0.742188, 0.742188]	5	3	2
[0.781250, 0.781250]	22	7	15
[0.820312, 0.820312]	7	2	5
[0.859375, 0.859375]	8	2	6
[0.878906, 0.878906]	1	0	1
[0.898438, 0.898438]	2	1	1

**Table 3:** Slice Thickness.

Slice Thickness [z] mm	Total	#Benign	#Malignant
1.25	24	4	20
2	68	24	44

**Table 4:** Results of the feature selection step using the point biserial correlation-hybrid-descriptive inferential (PBC-HID).

Group	Selection Algorithm	Selected Feature	Score
Non-harmonized	PBC-HID	original_gldm_ SmallDependenceHighGrayLevelEmphasis	0.38363
harmonized	PBC-HID	original_gldm_ SmallDependenceHighGrayLevelEmphasis	0.38669

**Table 5:** Results of the feature selection step using the LASSO algorithm.

Group	Selection Algorithm	Selected Features	Score
non-harmonized	LASSO	original_shape_Flatness original_gldm_ DependenceNonUniformityNormalized original_gldm_ SmallDependenceHighGrayLevelEmphasis original_glszm_ SizeZoneNonUniformity	/

**Table 6:** Results for a k-fold cross validation using the hybrid-descriptive inferential method.

Group	AUC	Accuracy	Sensitivity	Specificity
non-harmonized	0.76 ± 0.0218	0.73 ± 0.0085	0.22 ± 0.0135	0.94 ± 0.0068
harmonized	0.75 ± 0.0177	0.72 ± 0.0030	0.22 ± 0.0135	0.94 ± 0.0045

**Table 7:** Results for a k-fold stratified cross validation using the hybrid-descriptive inferential method.

Group	AUC	Accuracy	Sensitivity	Specificity
non-harmonized	0.75 ± 0.02	0.73 ± 0.0039	0.22 ± 0.000	0.94 ± 0.037
harmonized	0.75 ± 0.0131	0.72 ± 0.0058	0.22 ± 0.090	0.94 ± 0.0068

**Table 8:** Results for a K-Fold Stratified Cross Validation using the LASSO method.

Group	AUC	Accuracy	Sensitivity	Specificity
non-harmonized	0.74 ± 0.0162	0.73 ± 0.0069	0.28 ± 0.0202	0.92 ± 0.060

## Data Availability

Source code, documentation, and examples are available upon free request to the authors. After the publication of the article, the software will be available on the institutional websites of the authors (IBFM-CNR and Sapienza University).
